# Cognitive Radio-Assisted NOMA Broadcasting for 5G Cellular V2X Communications: Model of Roadside Unit Selection and SWIPT

**DOI:** 10.3390/s20061786

**Published:** 2020-03-24

**Authors:** Dinh-Thuan Do, Anh-Tu Le, Thi-Anh Hoang, Byung Moo Lee

**Affiliations:** 1 Wireless Communications Research Group, Faculty of Electrical & Electronics Engineering, Ton Duc Thang University, Ho Chi Minh City 700000, Vietnam; 2 Faculty of Electronics Technology, Industrial University of Ho Chi Minh City (IUH), Ho Chi Minh City 700000, Vietnam; leanhtu@iuh.edu.vn (A.-T.L.); hoangthianh@iuh.edu.vn (T.-A.H.); 3 School of Intelligent Mechatronics Engineering, Sejong University, Seoul 05006, Korea

**Keywords:** cognitive radio, NOMA, energy harvesting, vehicle to everything

## Abstract

The outage performance is a significant problem to implement the Cognitive Radio (CR) paradigm in the Vehicle to Everything (V2X) networks. Recently, more interest has focused on Non-Orthogonal Multiple Access (NOMA) in wireless-powered communication. In the conventional CR-enabled V2X-NOMA network, spectrum sensing and limited battery capacity at the Roadside Unit (RSU) may cause serious outage performance. In this study, RSU selection scheme is adopted. This paper presents an interesting model of a system with Simultaneous Wireless Information and Power Transfer (SWIPT) and a CR-enabled V2X-NOMA network. In the downlink, the RSU harvests wireless energy from Radio Frequency (RF) signals and senses the spectrum state at the same time. A CR-enabled V2X-NOMA system performance is presented by deriving exact expressions of outage probability of distant vehicles. In the overlay CR-enabled V2X-NOMA network, the constraints are transmit power and the number of designed RSU that make significant impacts on system performance. Simulation results show that the CR-enabled V2X-NOMA get benefits from energy harvesting and RSU selection scheme.

## 1. Introduction

Vehicle to everything (V2X) communications have been recently introduced with significant attention [1,2]. V2X networks provide the projected benefits such as reducing logistical costs for operating vehicular fleets, decreasing traffic-related fatalities, and introducing a variety of new business models [3,4]. To develop advanced techniques of 5G-enabled V2X networks in vehicular networks, coexistence of various kinds of V2X communications exists, and they may share the same wireless medium for data transmissions. In particular, vehicular to infrastructure (V2I) communications benefit vehicles that are associated with a central entity. Efficient and effective coordination of different types of V2X communications are analyzed in order to improve network performance under such a complex and heterogeneous topology as in [5,6,7,8]. The latest developments in the standardization of 802.11bd and V2X techniques are presented in [9].

### 1.1. Related Work

To cope with the increasing number of connected vehicles, one promising key candidate is allowing two vehicles to employ the same communication channel without increasing the spectrum requirement. In particular, non-orthogonal multiple access (NOMA) techniques are introduced as promising candidates for the future wireless communication vehicular networks [10,11]. In recent years, it has become popular to adopt NOMA in vehicular networks. In [12], the authors studied the NOMA in the V2X scenario in terms of capacity, and the difficulties in future generation communications are considered. By exploiting the principle of NOMA, two vehicles communicate with another vehicle through a vehicle-to-vehicle (V2V) link. In other way, a vehicle communicates with an RSU via a V2I and dedicated vehicles are known to have the ability of signal decoding adopted by successive interference cancellation (SIC). On the other hand, NOMA provides a new dimension for V2X services due to the capability of achieving high overloading transmission over limited resources. Therefore, implementing NOMA in V2X addresses the congestion situation, i.e., reducing the latency as transmission in a dense environment. In [13,14,15], NOMA-enabled 5G vehicular communication systems were studied to consider the key problems of interference management and handover. To enhance the spectral efficiency of the NOMA-enabled 5G vehicular communication system, the authors proposed a layered power control scheme to deploy joint optimization of power control and cell association, and then the traffic load conditions and the high mobility can be adapted. The authors in [16] studied a full duplex NOMA (FD-NOMA)-based decentralized V2X system model. In [17], the authors considered NOMA-based heterogeneous vehicular networks and proposed robust resource allocation to enhance both the throughput performance and reliability of such network. In the scenario reported in [18], the base station distributes time-frequency resources and employs semi-persistent scheduling in a non-orthogonal manner while a distributed power control scheme is adopted at the vehicles autonomously. It is confirmed that NOMA in a dense network can reduce the access latency and then the reliability can be improved.

Regarding other transmission techniques, namely cooperative relaying is introduced since it mitigates the effects of path-loss, shadowing, and multi-path fading [19]. Relay selection techniques in relaying network benefit to better signal are selected to serve destinations. Among those techniques, an opportunistic relay selection (ORS) is studied in [19]. The performance of near and far users can be improved when the partial relay selection (PRS) scheme is applied by selecting the best node among multiple intermediate relays [20,21]. In [20,21], the main performance metric is considered, such as the closed-form expressions of the outage probability. Through analyses and simulations, these relay-assisted cellular networks are considered as reasonable schemes to improve the coverage of 5G networks. In addition, the reduced outage probability can be achieved in an ORS-based network at the mode of delay-tolerant. Both the NOMA and orthogonal multiple access (OMA) transmission in the relay network are explored in [22]. This work introduced a buffer-aided relay selection scheme based on novel prioritization. The authors in [23] presented the two optimal relay selection (RS) schemes, namely max-weighted-harmonic-mean (MWHM) schemes and the two-stage weighted-max-min (WMM). Two RS schemes are implemented with cooperative NOMA with two modes of power allocations (fixed and adaptive) for the relays, and hence higher diversity transmission can be achieved.

It is hard to replace the battery and/or there is no power line for the secondary network containing the limited energy supply for the secondary users. Therefore, such situation limits performance of the secondary network. Fortunately, by harvesting the energy from the surrounding environments, wireless power can be obtained to address this problem. Radio frequency (RF) energy harvesting is applied in cognitive radio networks [24,25,26], which indicated that the energy signal can be harvested from the radio-frequency (RF) signals. Therefore, RF-based energy harvesting technique can provide flexible, sustainable and stable energy supply for wireless applications [27,28,29,30,31]. To provide battery charging, the primary network allows the secondary users dynamically decided to sense the spectrum or harvest the primary RF energy as in [28,29]. In other trends, throughput improvement in the secondary network can be achieved by utilizing both the optimal channel selection method and the harvested RF energy as in [30]. The energy consumption in cognitive radio sensor networks benefits from RF energy harvesting in [31]. The authors in [32] considered Simultaneous Wireless Information and Power Transfer (SWIPT) to implement cognitive radio with NOMA. In vehicular networks, the base station (BS) and roadside unit (RSU) in 5G-enabled networks are widely used facilities, especially in V2I communications. In particular, RSU presents a new way to provide high data-rate services and reliable connections to vehicles. An energy-efficient relay assisted transmission scheme is studied in the uplink cellular networks for V2X communications to provide delay insensitive applications [33]. The authors in [34] proposed a new approach for power allocation with energy efficiency optimization to employ in cellular device-to-device-assisted V2X communication network.

### 1.2. Our Contribution

The recent works have shown great potential of V2X communications with challenges such as energy efficiency, spectrum efficiency, dense vehicles, outage performance, and multiple services. Firstly, together with other techniques, NOMA is a key solution for the optimization of operation efficiency in the circumstance of crowded vehicles. Note that most of the papers focused on supporting low latency and high reliable services. For example, the authors in [35] considered the secure energy-efficient transmission in the context of SWIPT and vehicle-to-infrastructure (V2I) communication system with the imperfect channel state information (CSI). However, they did not apply the advantage of CR and NOMA. The coexistence of satellite and vehicular networks is further studied together with efficient resource utilization; however, the authors mainly investigated power allocation for the cognitive satellite-vehicular network in [36]. In fact, the inherent mobility of V2X-NOMA communications can be further considered to improve these metrics including outage performance, multiple services, network energy efficiency, and reliable transmission. Furthermore, a large number of vehicles need an infrastructure that is associated with a larger number of RSUs. With the rapid growth of infrastructure development, the energy consumption of RSU located everywhere is also a challenging issue. Meanwhile, most of the portable RSU only has a limited battery capacity due to size and space constraints, which directly affects users’ quality of experience (QoE). Thus, energy efficiency has become a key performance indicator for V2X-NOMA wireless networks. In particular, we derive an exact closed-form of the outage probability for each secondary destination under the context of CR. Such CR aims to further improve spectrum efficiency. In general, three folds would be the main benefits: (1) The SWIPT is proposed as a popular technique to prolong the operation duration of power-supply-limited devices in wireless networks. (2) CR-based V2X is considered as a special form of V2X, wherein the requirements of secondary and primary devices are strictly met so that performance improvement can be achieved. (3) The intermediate device can be selected to enhance the performance of related transmission. Those reasons motivate us to implement wireless power transfer to RSU to serve better transmission to vehicles and to overcome energy limitations at these low-cost RSUs.

The main contributions of this work are summarized as follows:

- It is the first work that formulates a situation in which many RSUs serve vehicles under operation of a CR-based V2X network. Such a V2X and NOMA scheme get benefits from the ability of energy harvesting to RSUs, which are devices with limited power source. The harvesting energy of each individual RSU then is selected to serve vehicles to achieve a minimum transmission rate and interference constraint. The link quality from the base station to vehicles is further benefitted by the RSU selection scheme.
- The outage probability metric is measured in exact expressions. The fixed power allocation factor is assigned to two vehicles. The power allocation factors and detection priority are considered as the main factors of different performance of two vehicles in terms of outage probability.
- Simulation results show that the outage performance achieved under the energy harvesting model may be affected by target rates, amount of harvested energy, and interference constraint. Moreover, simulation results show that there is a trade-off between the system performance and the power allocation factors assigned to vehicles.

The rest of this paper is organized as follows. In Section 2, the system model is presented. Section 3 presents the outage performance analysis. Section 4 presents simulation results. Finally, the paper is concluded in Section 5.

## 2. System Model

**Figure 1:**
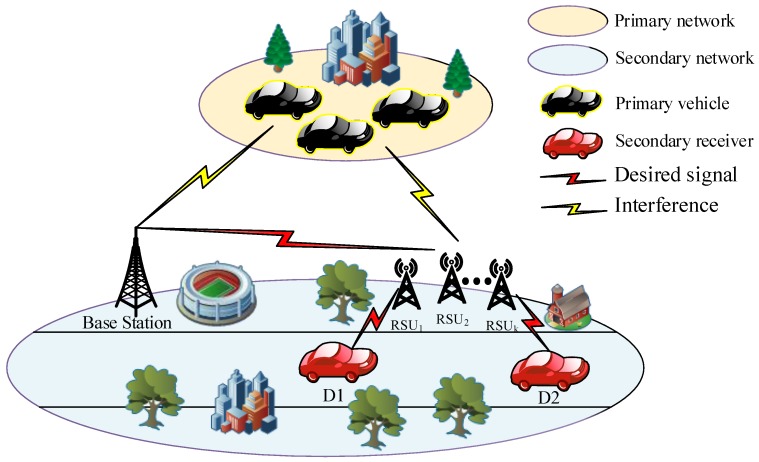
The model of the V2X-NOMA system.

We consider a V2X-NOMA depicted as in Figure 1. The CR architecture provides the downlink of secondary network from the base station (BS) to vehicles $D_{1},D_{2}$ under the support of many RSUs. The secondary network containing the BS and RSU can share the spectrum with the primary network without disturbing its normal communication. However, the BS can access the spectrum only when the primary network does not occupy that spectrum and such situation is associated with spectrum sensing. The RSUs are able to harvest wireless energy from the BS, shown in Figure 1. In the traditional scheme, the RSU directly transmits data to the vehicle; however, degraded performance occurs since vehicles move to several locations with a weak received signal. In this paper, the best RSU can be selected to serve the downlink. We denote $h_{{SR}_{k}}$ as the channel from the BS to *k*-th RSU. It is assumed that the primary and secondary networks employ channels with independent, flat, and block Rayleigh fading. This means that the system state will independently vary in different time slots and keep invariance in one time slot. The channel from the RSU to the primary receiver and the secondary vehicles are denoted by ${\left|h_{R_{k}P}\right|}^{2}$ and ${\left|h_{R_{k}D_{1}}\right|}^{2}$, respectively. The channel $h_{ab}$ for link from node *a* to node *b* follows an exponential distribution with parameters $\Omega_{ab}$.

To guarantee the operation of the considered network, the BS is required to opportunistically serve the vehicles with respect to securing the primary user’s QoS. The transmission procedure is occurred within two epochs in the considered system. In the first epoch, the BS sends the superimposed signal to the RSU node. To keep stable performance of the primary receiver, the transmission power of the BS is controlled. The interference power at the primary user is not allowed to exceed the maximum tolerable power level *I*, and, therefore, the power of the BS should satisfy

(1) $$P_{S}=\min\left\{P,\frac{I}{{\left|h_{SP}\right|}^{2}}\right\}$$

where *P* is the maximum transmission power of source in the primary network. It is worth noting that the interference from the primary network induces worse performance on the secondary network. Fortunately, interference can be reduced by adjusting an interference guard zone to each secondary user with a radius. This means that the interference from the primary network is dominated by the large scale path-loss. As a result, the interference from the primary network can be neglected for large distances [37].

NOMA signal at the BS, $x_{1},x_{2}$ are message signals dedicated to the first vehicle $V_{1}$ and the second vehicle $V_{2}$ and the corresponding power allocations are $a_{1},a_{2}$ with the constraint of $a_{1}>a_{2},a_{1}+a_{2}=1$. Regarding energy harvesting in terms of SWIPT, ρ is the power splitting factor in the power splitting based relaying protocol (0<ρ<1). It means that $P_{S}\left(1-\rho \right)$ corresponds to the power for signal processing from the BS to the RSU. Therefore, the received signal at the selected RSU is given by

(2) $$y_{R}=\left(\sqrt{a_{1}}x_{1}+\sqrt{a_{2}}x_{2}\right)\sqrt{P_{S}\left(1-\rho \right)h_{SR_{k}}}+n_{R},$$

where $n_{R}$ stands for the additive white Gaussian noise (AWGN), and it follows $\omega ∼CN\left(0,1\right)$. $h_{SR}$ is the gain of channel from the BS to *k*-th RSU.

At the RSU, $x_{1},x_{2}$ need to be decoded before forwarding to distant vehicles. As the assumption above, $a_{1}>a_{2}$ condition means that $x_{1}$ is decoded firstly, then subtracting $x_{1}$ from the received mixture signal to detect signal $x_{2}$ thanks to SIC. The signal-to-interference plus noise ratio (SINR), and signal-to-noise ratio (SNR) of the signal $x_{1},x_{2}$, respectively, at the RSU can be formulated by

(3) $$\gamma_{x_{1}}=\frac{a_{1}P_{S}\left(1-\rho \right){\left|h_{SR_{k}}\right|}^{2}}{a_{2}P_{S}\left(1-\rho \right){\left|h_{SR_{k}}\right|}^{2}+1}.$$

It is assumed that SIC is an ideal operation under the situation that several appropriate interference management techniques are included. We also consider imperfect SIC in other work. Due to perfect SIC, $x_{2}$ can be detected through the SNR below

(4) $$\gamma_{x_{2}}=a_{2}P_{S}\left(1-\rho \right){\left|h_{SR_{k}}\right|}^{2}.$$

**Remark** **1.** *In this system model, the transmission power at the RSU is able to be achieved once energy harvesting is enabled and signal detection is performed successfully at the RSU as well. Therefore, the transmission power levels for two signals $x_{1},x_{2}$ at the RSU are determined under the condition of successful detection of these signals $x_{1},x_{2}$. Then, we denote $P_{iR},i=1,2$ as transmission power at the RSU serving the first link and second link to $D_{1},D_{2}$, respectively.*

In the second phase, it is assumed that signals can be forwarded with new power allocation factors. To simplicity in notation, these factors are also $a_{1},a_{2}$. Thus, the received signal at each vehicle $D_{i}$ can be expressed as

(5) $$y_{Di}=\sqrt{P_{iR}}h_{R_{k}D_{i}}\left(\sqrt{a_{1}}x_{1}+\sqrt{a_{2}}x_{2}\right)+n_{Di},$$

where $n_{Di}$ represents AWGN at each receiving vehicle. $P_{1R}$ is transmission power at the RSU serving to $D_{1}$ and $P_{2R}$ is transmission power at the RSU serving to $D_{2}$.

The SINR at the vehicle $V_{1}$ when detecting the signal $x_{1}$ can be given as

(6) $$\gamma_{D,x_{1}}=\frac{a_{1}P_{R}{\left|h_{R_{k}D_{1}}\right|}^{2}}{a_{2}P_{1R}{\left|h_{R_{k}D_{1}}\right|}^{2}+1}$$

The SNR at $D_{2}$ when detecting the signal $x_{2}$ after performing SIC. It can be given as

(7) $$\gamma_{D,x_{2}}=a_{2}P_{R}{\left|h_{R_{k}D_{2}}\right|}^{2}$$

where $P_{R}$ is the transmission power at *R*.

The selection criterion of channel is considered to decide the best RSU. Then, the best RSU is used to forward the signal to a dedicated vehicle. The index of the RSU and corresponding channel gain are determined respectively by

(8) $$\begin{array}{c} k^{*}=\begin{array}{ccc} \arg & \max_{k=1,\ldots ,K} & {\left|h_{SR_{k}}\right|}^{2}, \end{array}\\ {\left|h_{SR_{k}*}\right|}^{2}=\begin{array}{cc} \max_{k=1,\ldots ,K} & {\left|h_{SR_{k}}\right|}^{2} \end{array} \end{array}$$

The CDF and PDF of such selected channel are formulated respectively from [38]

(9) $$\begin{array}{cc} F_{{\left|h_{SR_{k}}\right|}^{2}}\left(x\right) & ={\left(1-e^{-\frac{x}{\Omega_{SR}}}\right)}^{K}\\ \; & =1-\sum_{k=1}^{K}\left(\begin{array}{c} K\\ k \end{array}\right){\left(-1\right)}^{k-1}\left(e^{-\frac{kx}{\Omega_{SR}}}\right), \end{array}$$

(10) $$f_{{\left|h_{SR_{k}*}\right|}^{2}}\left(x\right)=K\sum_{k=0}^{K-1}\left(\begin{array}{c} K-1\\ k \end{array}\right)\frac{{\left(-1\right)}^{k}}{\Omega_{SR_{k}}}\exp\left(-\frac{\left(k+1\right)x}{\Omega_{SR_{k}}}\right)$$

## 3. Outage Performance Analysis

### 3.1. Outage Probability of $D_{1}$

In this section, the outage performance of each vehicle is investigated. It is equivalent to the probability of successful detection of signal $x_{1},x_{2}$ at these vehicles. In principle, the outage probability of a signal is defined as the probability that the achievable rate is below a threshold target rate. We denote $R_{1},R_{2}$ as a target rate for D1, D2, respectively. Such an outage probability for D1 is given by

(11) $$\begin{array}{c} P_{out\_D1}=\underset{A_{1}}{\underset{︸}{\mathrm{Pr}\left\{P_{S}{\left|h_{SR_{k}}\right|}^{2}<\delta \right\}}}+\underset{A_{2}}{\underset{︸}{\mathrm{Pr}\left\{P_{S}{\left|h_{SR_{k}}\right|}^{2}>\delta ,P_{1R}{\left|h_{R_{k}D_{1}}\right|}^{2}<\delta \right\}}}, \end{array}$$

where $P_{S}=\min\left\{P,\frac{I}{{\left|h_{SP}\right|}^{2}}\right\}$, $\delta =\frac{\varpi_{1}}{a_{1}-\varpi_{1}a_{2}}$, in which $\varpi_{1}=2^{2R_{1}}-1$.

In this situation, the transmission power obtained at the RSU $P_{1R}$ after performing energy harvesting can be determined as the following analysis. It is worth noting that the RSU can detect the message successfully from the BS if the following condition occurs:

(12) $$\frac{1}{2}\log\left(1+\frac{a_{1}P_{S}\left(1-\rho \right){\left|h_{SR_{k}}\right|}^{2}}{a_{2}P_{S}\left(1-\rho \right){\left|h_{SR_{k}}\right|}^{2}+1}\right)⩾R_{1}$$

Thus, the power splitting factor $\rho_{1}⩽1-\frac{\varpi_{1}}{\left(a_{1}-\varpi_{1}a_{2}\right)P_{S}{\left|h_{SR_{k}}\right|}^{2}}$, and $R_{1}$ denotes the targeted data rate. $\rho_{1,\max}⩽1-\frac{\varpi_{1}}{\left(a_{1}-\varpi_{1}a_{2}\right)P_{S}{\left|h_{SR_{k}}\right|}^{2}}$.

The harvested power at the RSU $P_{1R}^{\prime }$ is given by [11]

(13) $$P_{1R}^{\prime }=\eta P_{S}{\left|h_{SR_{k}}\right|}^{2}\rho_{1,\max}=\left\{\begin{array}{c} \eta \left(P_{S}{\left|h_{SR_{k}}\right|}^{2}-\delta \right),\begin{array}{cc} if & P_{S}{\left|h_{SR_{k}}\right|}^{2}>\delta \end{array}\\ \begin{array}{cc} 0, & otherwise, \end{array} \end{array}\right.$$

where 0<η≤1 denotes the energy efficiency in energy harvesting protocol.

To guarantee operation of the primary network, the transmission power at the RSU must be constrained by

(14) $$P_{1R}=\min\left\{P_{1R}^{\prime },\frac{I}{{\left|h_{R_{k}P}\right|}^{2}}\right\}$$

**Lemma** **1.** *The probability $\mathrm{Pr}\left\{P_{S}{\left|h_{SR_{k}}\right|}^{2}<\delta \right\}$ can be calculated by [19]*

(15) $$\begin{array}{cc} A_{1} & =\left[1-\sum_{k=1}^{K}\left(\begin{array}{c} K\\ k \end{array}\right){\left(-1\right)}^{k-1}\exp\left(-\frac{\delta k}{P\Omega_{SR}}\right)\right]\left(1-\exp\left(-\frac{I}{P\Omega_{SP}}\right)\right)\\ \; & +\exp\left(-\frac{I}{P\Omega_{SP}}\right)-\sum_{k=1}^{K}\left(\begin{array}{c} K\\ k \end{array}\right)\frac{I\Omega_{SR}{\left(-1\right)}^{k-1}}{I\Omega_{SR}+\delta k\Omega_{SP}}\exp\left(-\frac{\delta k\Omega_{SP}+I\Omega_{SR}}{P\Omega_{SR}\Omega_{SP}}\right) \end{array}$$

**Proof.**  See in Appendix A.  □

Next, the term $A_{2}$ can be characterized as below:

(16) $$\begin{array}{cc} A_{2} & =\left(\underset{A_{21}}{\underset{︸}{\mathrm{Pr}\left\{P{\left|h_{SR_{k}}\right|}^{2}>\delta ,{\left|h_{R_{k}D_{1}}\right|}^{2}<\frac{\delta }{\eta \left(P{\left|h_{SR_{k}}\right|}^{2}-\delta \right)},{\left|h_{R_{k}P}\right|}^{2}<\frac{I}{\eta \left(P{\left|h_{SR_{k}}\right|}^{2}-\delta \right)}\right\}}}\right.\\ \; & +\left.\underset{A_{22}}{\underset{︸}{\mathrm{Pr}\left\{P{\left|h_{SR_{k}}\right|}^{2}>\delta ,{\left|h_{R_{k}D_{1}}\right|}^{2}<\frac{\delta {\left|h_{R_{k}P}\right|}^{2}}{I},{\left|h_{R_{k}P}\right|}^{2}>\frac{I}{\eta \left(P{\left|h_{SR_{k}}\right|}^{2}-\delta \right)}\right\}}}\right)\mathrm{Pr}\left\{{\left|h_{SP}\right|}^{2}<\frac{I}{P}\right\}\\ \; & +\underset{A_{23}}{\underset{︸}{\mathrm{Pr}\left\{\frac{I{\left|h_{SR_{k}}\right|}^{2}}{{\left|h_{SP}\right|}^{2}}>\delta ,{\left|h_{SP}\right|}^{2}>\frac{I}{P},{\left|h_{R_{k}D_{1}}\right|}^{2}<\frac{\delta }{\eta \left(\frac{I{\left|h_{SR_{k}}\right|}^{2}}{{\left|h_{SP}\right|}^{2}}-\delta \right)},{\left|h_{R_{k}P}\right|}^{2}<\frac{I}{\eta \left(\frac{I{\left|h_{SR_{k}}\right|}^{2}}{{\left|h_{SP}\right|}^{2}}-\delta \right)}\right\}}}\\ \; & +\underset{A_{24}}{\underset{︸}{\mathrm{Pr}\left\{\frac{I{\left|h_{SR_{k}}\right|}^{2}}{{\left|h_{SP}\right|}^{2}}>\delta ,{\left|h_{SP}\right|}^{2}>\frac{I}{P},{\left|h_{R_{k}D_{1}}\right|}^{2}<\frac{\delta {\left|h_{R_{k}P}\right|}^{2}}{I},{\left|h_{R_{k}P}\right|}^{2}>\frac{I}{\eta \left(\frac{I{\left|h_{SR_{k}}\right|}^{2}}{{\left|h_{SP}\right|}^{2}}-\delta \right)}\right\}}} \end{array}$$

Firstly, $A_{21}$ can be computed by

(17) $$\begin{array}{cc} A_{21} & =\mathrm{Pr}\left\{{\left|h_{SR_{k}}\right|}^{2}>\frac{\delta }{P},{\left|h_{R_{k}D_{1}}\right|}^{2}<\frac{\delta }{\eta \left(P{\left|h_{SR_{k}}\right|}^{2}-\delta \right)},{\left|h_{R_{k}P}\right|}^{2}<\frac{I}{\eta \left(P{\left|h_{SR_{k}}\right|}^{2}-\delta \right)}\right\}\\ \; & =\int_{\frac{\delta }{P}}^{\infty }F_{{\left|h_{R_{k}D_{1}}\right|}^{2}}\left(\frac{\delta }{\eta \left(Px-\delta \right)}\right)F_{{\left|h_{R_{k}P}\right|}^{2}}\left(\frac{I}{\eta \left(Px-\delta \right)}\right)f_{{\left|h_{SR_{k}}\right|}^{2}}\left(x\right)dx\\ \; & =\sum_{k=1}^{K}\left(\begin{array}{c} K\\ k \end{array}\right){\left(-1\right)}^{k-1}\exp\left(-\frac{\delta k}{P\Omega_{SR}}\right)\left(1-2\sqrt{\frac{Ik}{P\eta \Omega_{RP}\Omega_{SR}}}K_{1}\left(2\sqrt{\frac{Ik}{\eta P\Omega_{RP}\Omega_{SR}}}\right)\right.\\ \; & -2\sqrt{\frac{\delta k}{\eta P\Omega_{RD_{1}}\Omega_{SR}}}K_{1}\left(2\sqrt{\frac{\delta k}{\eta P\Omega_{RD_{1}}\Omega_{SR}}}\right)\\ \; & +\left.2\sqrt{\frac{\left(\delta \Omega_{RP}+I\Omega_{RD_{1}}\right)k}{\Omega_{RD_{1}}P\eta \Omega_{RP}\Omega_{SR}}}K_{1}\left(2\sqrt{\frac{\left(\delta \Omega_{RP}+I\Omega_{RD_{1}}\right)k}{\Omega_{RD_{1}}\eta \Omega_{RP}P\Omega_{SR}}}\right)\right) \end{array}$$

**Lemma** **2.** *The term $A_{22}$ can be calculated as [20]*

(18) $$\begin{array}{cc} A_{22} & =2\sum_{k=1}^{K}\left(\begin{array}{c} K\\ k \end{array}\right){\left(-1\right)}^{k-1}e^{-\frac{k\delta }{\Omega_{SR}P}}\left(\sqrt{\frac{Ik}{\Omega_{RP}\Omega_{SR}P\eta }}K_{1}\left(2\sqrt{\frac{Ik}{\Omega_{RP}\Omega_{SR}P\eta }}\right)\right.\\ \; & -\left.\frac{I\Omega_{RD_{1}}}{\delta \Omega_{RP}+I\Omega_{R_{k}D_{1}}}\sqrt{\frac{\left(\delta \Omega_{RP}+I\Omega_{RD_{1}}\right)k}{\Omega_{RD_{1}}\Omega_{RP}\Omega_{SR}P\eta }}K_{1}\left(2\sqrt{\frac{\left(\delta \Omega_{RP}+I\Omega_{RD_{1}}\right)k}{\Omega_{RD_{1}}\Omega_{RP}\Omega_{SR}P\eta }}\right)\right). \end{array}$$

**Proof.**  See in Appendix B.

Similarly, $A_{23}$ can be calculated as

(19) $$\begin{array}{c} A_{23}=\sum_{k=1}^{K}\left(\begin{array}{c} K\\ k \end{array}\right){\left(-1\right)}^{k-1}\frac{I\Omega_{SR}}{I\Omega_{SR}+\Omega_{SP}\delta k}\exp\left(-\frac{I\Omega_{SR}+\Omega_{SP}\delta k}{P\Omega_{SP}\Omega_{SR}}\right)\\ -\frac{1}{\Omega_{SP}}\sum_{k=1}^{K}\left(\begin{array}{c} K\\ k \end{array}\right){\left(-1\right)}^{k-1}\int_{\frac{I}{P}}^{\infty }\left(2\sqrt{\frac{k}{\eta \Omega_{RP}\Omega_{SR}}y}K_{1}\left(2\sqrt{\frac{k}{\eta \Omega_{RP}\Omega_{SR}}y}\right)\exp\left(-\left(\frac{I\Omega_{SR}+\Omega_{SP}\delta k}{I\Omega_{SP}\Omega_{SR}}\right)y\right)\right.\\ +2\sqrt{\frac{\delta k}{\eta I\Omega_{RD_{1}}\Omega_{SR}}y}K_{1}\left(2\sqrt{\frac{\delta ky}{\eta I\Omega_{SR}\Omega_{RD_{1}}}y}\right)\exp\left(-\left(\frac{I\Omega_{SR}+\Omega_{SP}\delta k}{I\Omega_{SP}\Omega_{SR}}y\right)\right)\\ \left.-2\sqrt{\frac{\left(\delta \Omega_{RP}+I\Omega_{RD_{1}}\right)ky}{\eta I\Omega_{RD_{1}}\Omega_{RP}\Omega_{SR}}}K_{1}\left(2\sqrt{\frac{\left(\delta \Omega_{RP}+I\Omega_{RD_{1}}\right)ky}{\eta I\Omega_{SR}\Omega_{RD_{1}}\Omega_{RP}}}\right)\exp\left(-\left(\frac{I\Omega_{SR}+\Omega_{SP}\delta k}{I\Omega_{SP}\Omega_{SR}}\right)y\right)\right)dy \end{array}$$

Performing similar computation, $A_{24}$ is given by

(20) $$\begin{array}{c} A_{24}=\frac{2}{\Omega_{SP}}\sum_{k=1}^{K}\left(\begin{array}{c} K\\ k \end{array}\right){\left(-1\right)}^{k-1}\int_{\frac{I}{P}}^{\infty }\left(\sqrt{\frac{ky}{\Omega_{RP}\Omega_{SR}\eta }}K_{1}\left(2\sqrt{\frac{ky}{\Omega_{RP}\Omega_{SR}\eta }}\right)e^{-\left(\frac{I\Omega_{SR}+\Omega_{SP}k\delta }{I\Omega_{SR}\Omega_{SP}}\right)y}\right.\\ \left.-\frac{I\Omega_{RD_{1}}}{\Omega_{RP}\delta +I\Omega_{RD_{1}}}\sqrt{\frac{\left(\Omega_{RP}\delta +I\Omega_{RD_{1}}\right)ky}{\Omega_{RD_{1}}\Omega_{RP}\Omega_{SR}\eta I}}K_{1}\left(2\sqrt{\frac{\left(\Omega_{RP}\delta +I\Omega_{RD_{1}}\right)ky}{\Omega_{RD_{1}}\Omega_{RP}\Omega_{SR}\eta I}}\right)e^{-\left(\frac{I\Omega_{SR}+\Omega_{SP}k\delta }{I\Omega_{SR}\Omega_{SP}}\right)y}\right)dy \end{array}$$

It is noted that the probability of channel between the primary network and the secondary network is given as

(21) $$\mathrm{Pr}\left\{{\left|h_{SP}\right|}^{2}<\frac{I}{P}\right\}=1-\exp\left(-\frac{I}{P\Omega_{SP}}\right)$$

Substituting Equations (17)–(20) into (16), $A_{2}$ can be calculated by

(22) $$A_{2}=\left(A_{21}+A_{22}\right)\left(1-\exp\left(-\frac{I}{P\Omega_{SP}}\right)\right)+A_{23}+A_{24}.$$

Then, $P_{out\_D1}$ can be computed based on $A_{1},A_{2}$.

### 3.2. Outage Probability of $D_{2}$

Similar to the outage probability related to the signal $x_{1}$, the outage probability of the signal $x_{2}$ at the second vehicle can be expressed as

(23) $$P_{out\_D2}=\underset{B_{1}}{\underset{︸}{\mathrm{Pr}\left\{P_{S}{\left|h_{SR_{k}}\right|}^{2}<\gamma \right\}}}+\underset{B_{2}}{\underset{︸}{\mathrm{Pr}\left\{P_{S}{\left|h_{SR_{k}}\right|}^{2}>\gamma ,P_{2R}{\left|h_{R_{k}D_{2}}\right|}^{2}<\gamma \right\}}},$$

where $\gamma =\frac{\varpi_{2}}{a_{2}}$.

To determine the transmission power at RSU to vehicle $D_{2}$, we consider the ability of successful detection at RSU for signal $x_{2}$ as

(24) $$\frac{1}{2}\log\left(1+a_{2}P_{S}\left(1-\rho \right){\left|h_{SR_{k}}\right|}^{2}\right)⩾R_{2}$$

The power splitting factor $\rho_{2}⩽1-\frac{\varpi_{2}}{a_{2}P_{S}{\left|h_{SR_{k}}\right|}^{2}}$, where $\varpi_{2}=2^{2R_{2}}-1$ and $R_{2}$ denotes the target rate of the second vehicle $\rho_{2,\max}⩽1-\frac{\varpi_{2}}{a_{2}P_{S}{\left|h_{SR_{k}}\right|}^{2}}$.

Therefore, the harvested power can be obtained at the RSU after energy harvesting is given by

(25) $$P_{2R}^{\prime }=\eta P_{S}{\left|h_{SR_{k}}\right|}^{2}\rho_{2,\max}=\left\{\begin{array}{c} \eta \left(P_{S}{\left|h_{SR_{k}}\right|}^{2}-\gamma \right),\begin{array}{cc} if & P_{S}{\left|h_{SR_{k}}\right|}^{2}>\gamma \end{array}\\ \begin{array}{cc} 0, & otherwise. \end{array} \end{array}\right.$$

To keep cognitive radio network works, the transmission power at the RSU for the second link from the RSU to the second vehicle must be constrained by

(26) $$P_{2R}=\min\left\{P_{2R}^{\prime },\frac{I}{{\left|h_{R_{k}P}\right|}^{2}}\right\}$$

We then continue to compute $B_{1}$ as below:

(27) $$\begin{array}{cc} B_{1} & =\left[1-\sum_{k=1}^{K}\left(\begin{array}{c} K\\ k \end{array}\right){\left(-1\right)}^{k-1}\exp\left(-\frac{\gamma k}{P\Omega_{SR}}\right)\right]\left(1-\exp\left(-\frac{I}{P\Omega_{SP}}\right)\right)\\ \; & +\exp\left(-\frac{I}{P\Omega_{SP}}\right)-\sum_{k=1}^{K}\left(\begin{array}{c} K\\ k \end{array}\right)\frac{I\Omega_{SR}{\left(-1\right)}^{k-1}}{I\Omega_{SR}+\gamma k\Omega_{SP}}\exp\left(-\frac{\gamma k\Omega_{SP}+I\Omega_{SR}}{P\Omega_{SR}\Omega_{SP}}\right) \end{array}$$

In addition, $B_{2}$ can be rewritten as

(28) $$\begin{array}{cc} B_{2} & =\mathrm{Pr}\left\{P_{S}{\left|h_{SR_{k}}\right|}^{2}>\gamma ,P_{2R}{\left|h_{R_{k}D_{2}}\right|}^{2}<\gamma \right\}\\ \; & =\mathrm{Pr}\left\{P_{S}{\left|h_{SR_{k}}\right|}^{2}>\gamma ,P_{2r}^{\prime }{\left|h_{R_{k}D_{2}}\right|}^{2}<\gamma ,P_{2R}^{\prime }<\frac{I}{{\left|h_{R_{k}P}\right|}^{2}}\right\}\\ \; & +\mathrm{Pr}\left\{P_{S}{\left|h_{SR_{k}}\right|}^{2}>\gamma ,P_{2r}^{\prime }{\left|h_{R_{k}D_{2}}\right|}^{2}<\gamma ,P_{2R}^{\prime }>\frac{I}{{\left|h_{R_{k}P}\right|}^{2}}\right\} \end{array}$$

To compute $B_{2}$, it can be extended as

(29) $$\begin{array}{cc} B_{2} & =\left(\underset{B_{21}}{\underset{︸}{\mathrm{Pr}\left\{P{\left|h_{SR_{k}}\right|}^{2}>\gamma ,{\left|h_{R_{k}D_{2}}\right|}^{2}<\frac{\gamma }{\eta \left(P{\left|h_{SR_{k}}\right|}^{2}-\gamma \right)},{\left|h_{R_{k}P}\right|}^{2}<\frac{I}{\eta \left(P{\left|h_{SR_{k}}\right|}^{2}-\gamma \right)}\right\}}}\right.\\ & +\left.\underset{B_{22}}{\underset{︸}{\mathrm{Pr}\left\{P{\left|h_{SR_{k}}\right|}^{2}>\gamma ,{\left|h_{R_{k}D_{2}}\right|}^{2}<\frac{\gamma {\left|h_{R_{k}P}\right|}^{2}}{I},{\left|h_{R_{k}P}\right|}^{2}>\frac{I}{\eta \left(P{\left|h_{SR_{k}}\right|}^{2}-\gamma \right)}\right\}}}\right)\mathrm{Pr}\left\{{\left|h_{SP}\right|}^{2}<\frac{I}{P}\right\}\\ & +\underset{B_{23}}{\underset{︸}{\mathrm{Pr}\left\{\frac{I{\left|h_{SR_{k}}\right|}^{2}}{{\left|h_{SP}\right|}^{2}}>\gamma ,{\left|h_{SP}\right|}^{2}>\frac{I}{P},{\left|h_{R_{k}D_{2}}\right|}^{2}<\frac{\gamma }{\eta \left(\frac{I{\left|h_{SR_{k}}\right|}^{2}}{{\left|h_{SP}\right|}^{2}}-\gamma \right)},{\left|h_{R_{k}P}\right|}^{2}<\frac{I}{\eta \left(\frac{I{\left|h_{SR_{k}}\right|}^{2}}{{\left|h_{SP}\right|}^{2}}-\gamma \right)}\right\}}}\\ & +\underset{B_{24}}{\underset{︸}{\mathrm{Pr}\left\{\frac{I{\left|h_{SR_{k}}\right|}^{2}}{{\left|h_{SP}\right|}^{2}}>\gamma ,{\left|h_{SP}\right|}^{2}>\frac{I}{P},{\left|h_{R_{k}D_{2}}\right|}^{2}<\frac{\gamma {\left|h_{R_{k}P}\right|}^{2}}{I},{\left|h_{R_{k}P}\right|}^{2}>\frac{I}{\eta \left(\frac{I{\left|h_{SR_{k}}\right|}^{2}}{{\left|h_{SP}\right|}^{2}}-\gamma \right)}\right\}}}. \end{array}$$

It is noted that the first term $B_{21}$ can be calculated as

(30) $$\begin{array}{c} B_{21}=\sum_{k=1}^{K}\left(\begin{array}{c} K\\ k \end{array}\right){\left(-1\right)}^{k-1}\exp\left(-\frac{\gamma k}{P\Omega_{SR}}\right)\left(1-2\sqrt{\frac{Ik}{P\eta \Omega_{RP}\Omega_{SR}}}K_{1}\left(2\sqrt{\frac{Ik}{\eta P\Omega_{RP}\Omega_{SR}}}\right)\right.\\ \left.-2\sqrt{\frac{\gamma k}{\eta P\Omega_{RD_{2}}\Omega_{SR}}}K_{1}\left(2\sqrt{\frac{\gamma k}{\eta P\Omega_{RD_{2}}\Omega_{SR}}}\right)+2\sqrt{\frac{\left(\gamma \Omega_{RP}+I\Omega_{RD_{2}}\right)k}{\Omega_{RD_{2}}P\eta \Omega_{RP}\Omega_{SR}}}K_{1}\left(2\sqrt{\frac{\left(\gamma \Omega_{RP}+I\Omega_{RD_{2}}\right)k}{\Omega_{RD_{2}}\eta \Omega_{RP}P\Omega_{SR}}}\right)\right). \end{array}$$

The second term $B_{22}$ can be expressed by

(31) $$\begin{array}{cc} B_{22} & =2\sum_{k=1}^{K}\left(\begin{array}{c} K\\ k \end{array}\right){\left(-1\right)}^{k-1}e^{-\frac{k\gamma }{\Omega_{SR}P}}\left(\sqrt{\frac{Ik}{\Omega_{RP}\Omega_{SR}P\eta }}K_{1}\left(2\sqrt{\frac{Ik}{\Omega_{RP}\Omega_{SR}P\eta }}\right)\right.\\ \; & -\left.\frac{I\Omega_{RD_{2}}}{\gamma \Omega_{RP}+I\Omega_{RD_{2}}}\sqrt{\frac{\left(\gamma \Omega_{RP}+I\Omega_{RD_{2}}\right)k}{\Omega_{RD_{2}}\Omega_{RP}\Omega_{SR}P\eta }}K_{1}\left(2\sqrt{\frac{\left(\gamma \Omega_{RP}+I\Omega_{RD_{2}}\right)k}{\Omega_{RD_{2}}\Omega_{RP}\Omega_{SR}P\eta }}\right)\right) \end{array}$$

The third term $B_{23}$ can be calculated as

(32) $$\begin{array}{cc} B_{23} & =\mathrm{Pr}\left\{\frac{{\left|h_{SR_{k}}\right|}^{2}}{{\left|h_{SP}\right|}^{2}}>\frac{\delta }{I},{\left|h_{SP}\right|}^{2}>\frac{I}{P},{\left|h_{R_{k}D_{2}}\right|}^{2}<\frac{\delta }{\eta \left(\frac{I{\left|h_{SR_{k}}\right|}^{2}}{X_{SP}}-\delta \right)},{\left|h_{RP}\right|}^{2}<\frac{I}{\eta \left(\frac{{\left|h_{SR_{k}}\right|}^{2}}{X_{SP}}I-\delta \right)}\right\}\\ \; & =\int_{\frac{I}{P}}^{\infty }\int_{\frac{\delta y}{I}}^{\infty }F_{{\left|h_{R_{k}D_{2}}\right|}^{2}}\left(\frac{\delta }{\eta \left(\frac{Ix}{y}-\delta \right)}\right)F_{{\left|h_{SP}\right|}^{2}}\left(\frac{I}{\eta \left(\frac{Ix}{y}-\delta \right)}\right)f_{{\left|h_{SR_{k}}\right|}^{2}}\left(x\right)dxf_{{\left|h_{SP}\right|}^{2}}\left(y\right)dy \end{array}$$

Similarly, $B_{23}$ is rewritten as

(33) $$\begin{array}{cc} B_{23} & =\sum_{k=1}^{K}\left(\begin{array}{c} K\\ k \end{array}\right){\left(-1\right)}^{k-1}\frac{I\Omega_{SR}}{I\Omega_{SR}+\Omega_{SP}\delta k}\exp\left(-\frac{I\Omega_{SR}+\Omega_{SP}\gamma k}{P\Omega_{SP}\Omega_{SR}}\right)\\ & -\frac{1}{\Omega_{SP}}\sum_{k=1}^{K}\left(\begin{array}{c} K\\ k \end{array}\right){\left(-1\right)}^{k-1}\int_{\frac{I}{P}}^{\infty }\left(2\sqrt{\frac{k}{\eta \Omega_{RP}\Omega_{SR}}y}K_{1}\left(2\sqrt{\frac{k}{\eta \Omega_{RP}\Omega_{SR}}y}\right)\exp\left(-\left(\frac{I\Omega_{SR}+\Omega_{SP}\gamma k}{I\Omega_{SP}\Omega_{SR}}\right)y\right)\right.\\ & +2\sqrt{\frac{\gamma k}{\eta I\Omega_{RD_{2}}\Omega_{SR}}y}K_{1}\left(2\sqrt{\frac{\gamma ky}{\eta I\Omega_{SR}\Omega_{RD_{2}}}y}\right)\exp\left(-\left(\frac{I\Omega_{SR}+\Omega_{SP}\gamma k}{I\Omega_{SP}\Omega_{SR}}y\right)\right)\\ & -\left.2\sqrt{\frac{\left(\gamma \Omega_{RP}+I\Omega_{RD_{2}}\right)ky}{\eta I\Omega_{RD_{2}}\Omega_{RP}\Omega_{SR}}}K_{1}\left(2\sqrt{\frac{\left(\gamma \Omega_{RP}+I\Omega_{RD_{2}}\right)ky}{\eta I\Omega_{SR}\Omega_{RD_{2}}\Omega_{RP}}}\right)\exp\left(-\left(\frac{I\Omega_{SR}+\Omega_{SP}\gamma k}{I\Omega_{SP}\Omega_{SR}}\right)y\right)\right)dy \end{array}$$

We then compute $A_{24}$ as

(34) $$\begin{array}{cc} B_{24} & =\mathrm{Pr}\left\{\frac{{\left|h_{SR_{k}}\right|}^{2}}{{\left|h_{SP}\right|}^{2}}>\delta ,{\left|h_{SP}\right|}^{2}>\frac{I}{P},{\left|h_{R_{k}D_{2}}\right|}^{2}<\frac{\delta {\left|h_{RP}\right|}^{2}}{I},{\left|h_{RP}\right|}^{2}>\frac{I}{\eta \left(\frac{{\left|h_{SR_{k}}\right|}^{2}I}{{\left|h_{SP}\right|}^{2}}-\delta \right)}\right\}\\ & =\int_{\frac{I}{P}}^{\infty }f_{{\left|h_{SP}\right|}^{2}}\left(y\right)\int_{\frac{\delta y}{I}}^{\infty }f_{{\left|h_{SR_{k}}\right|}^{2}}\left(x\right)\int_{\frac{I}{\eta \left(\frac{I}{y}x-\delta \right)}}^{\infty }f_{{\left|h_{RP}\right|}^{2}}\left(z\right)F_{{\left|h_{R_{k}D_{2}}\right|}^{2}}\left(\frac{\delta z}{I}\right)dzdxdy\\ & =\frac{2}{\Omega_{SP}}\sum_{k=1}^{K}\left(\begin{array}{c} K\\ k \end{array}\right){\left(-1\right)}^{k-1}\int_{\frac{I}{P}}^{\infty }\left(\sqrt{\frac{ky}{\Omega_{RP}\Omega_{SR}\eta }}K_{1}\left(2\sqrt{\frac{ky}{\Omega_{RP}\Omega_{SR}\eta }}\right)e^{-\left(\frac{I\Omega_{SR}+\Omega_{SP}k\delta }{I\Omega_{SR}\Omega_{SP}}\right)y}\right.\\ & -\left.\frac{I\Omega_{RD_{2}}}{\Omega_{RP}\delta +I\Omega_{RD_{2}}}\sqrt{\frac{\left(\Omega_{RP}\delta +I\Omega_{RD_{2}}\right)ky}{\Omega_{RD_{2}}\Omega_{RP}\Omega_{SR}\eta I}}K_{1}\left(2\sqrt{\frac{\left(\Omega_{RP}\delta +I\Omega_{RD_{2}}\right)ky}{\Omega_{RD_{2}}\Omega_{RP}\Omega_{SR}\eta I}}\right)e^{-\left(\frac{I\Omega_{SR}+\Omega_{SP}k\delta }{I\Omega_{SR}\Omega_{SP}}\right)y}\right)dy \end{array}$$

We continue to use the fact that $\mathrm{Pr}\left\{{\left|h_{SP}\right|}^{2}<\frac{I}{P}\right\}=1-\exp\left(-\frac{I}{P\Omega_{SP}}\right)$; then, $B_{2}$ is given as

(35) $$B_{2}=\left(B_{21}+B_{22}\right)\left(1-\exp\left(-\frac{I}{P\Omega_{SP}}\right)\right)+B_{23}+B_{24}.$$

## 4. Numerical Results

This section provides numerical and simulation results in terms of the outage probability over Rayleigh fading channels. We run $10^{6}$ iterations for Monte Carlo simulation results. We try to simulate with a high number of trials to provide the simulation results more precisely and and hence analytical results are closer to the theoretical results. Our results depend on the iterations of a Monte Carlo simulation where the sample mean is computed, followed by the population mean, and then calculate a 95% confidence interval for each observation. In particular, we set power allocation factors as $a_{1}=0.8$, $a_{2}=0.2$, the channel gains $\Omega_{SR}=5$, $\Omega_{SP}=\Omega_{RP}=0.1$, $\Omega_{RD_{1}}=1$, $\Omega_{RD_{2}}=2$, I=20 dB, $R_{1}=R_{2}=1$ (bits/s/Hz), η=0.9 and K=1 except for specific cases.

**Figure 2:**
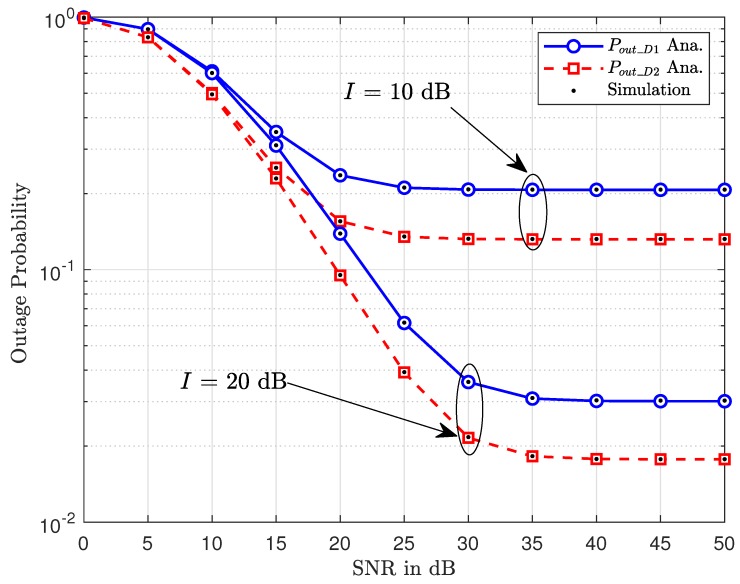
Outage performance of two vehicles versus SNR at the BS with different *I*.

Figure 2 shows the outage performance related to the ability of detecting $x_{1},x_{2}$ at $D_{1},D_{2}$, respectively. It can be seen that, when increasing SNR at the BS, a higher level of power is used to signal processing, then outage improvement is archived, and hence significantly low outage performance at a high SNR regime is achieved. When we set I= 20 (dB), the outage performance of two vehicles are better than the case of I=10 (dB). The other observation is that the outage of the second vehicle shows better performance than that of the first vehicle and the performance gap is clearer at high SNR values (SNR is greater than 20 dB). The reason is that $D_{2}$ eliminates interference from $D_{1}$ by deploying SIC, so SNR achieved at $D_{2}$ is higher than at $D_{1}$. It can be further explained that $D_{1}$ detects the desired signal without eliminating interference from other vehicles that makes the probability worse. Moreover, the simulation results by using Monte Carlo match very well with the analytical ones. This confirms the correctness of the derived closed-form expressions related to outage behavior of two vehicles.

**Figure 3:**
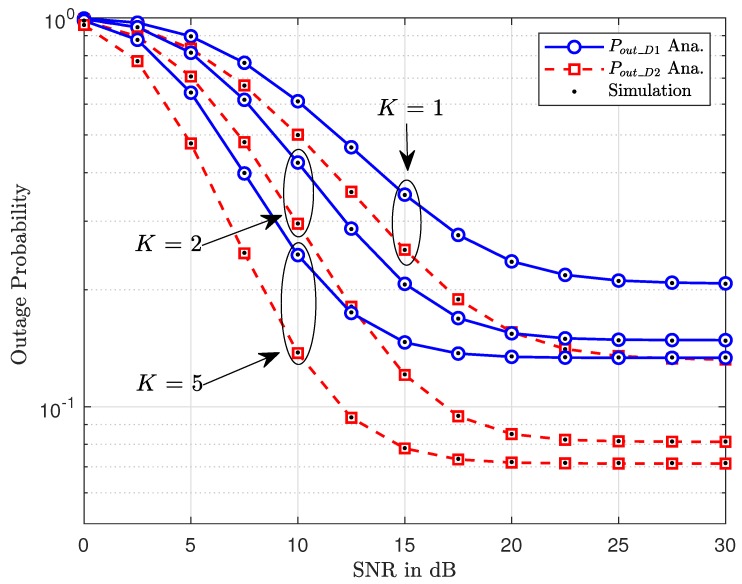
Outage performance of two vehicles versus SNR at the BS with different *K*.

Figure 3 compares the outage performance of two vehicles for different values of the number of RSUs. It can be observed from the same figure that $D_{2}$ achieves a smaller outage probability compared with that of $D_{1}$ regardless of any number of RSU. K=5 exhibits as the best performance to indicate a role selecting a better signal among larger numbers of RSU. Figure 3 also shows that, although better outage can be obtained by increasing SNR from 0 (dB) to 20 (dB), an increase in SNR at the BS over this point (20 dB) still meets the saturation of outage behavior at a high region of SNR. Interestingly, a larger performance gap among two vehicles can be seen clearly at high SNR. The main reason is that different conditions of signal detection and different power allocation factors are assigned to each signal.

**Figure 4:**
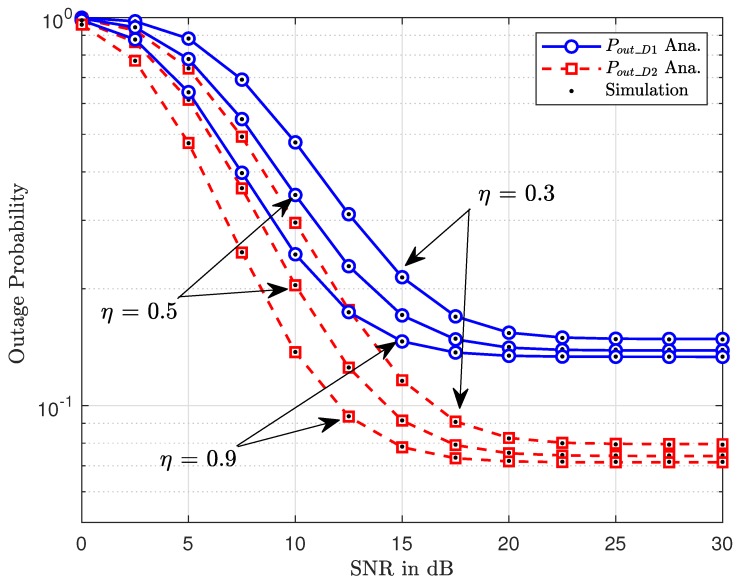
Impact of percentage of harvested power at RSU on outage performance.

**Figure 5:**
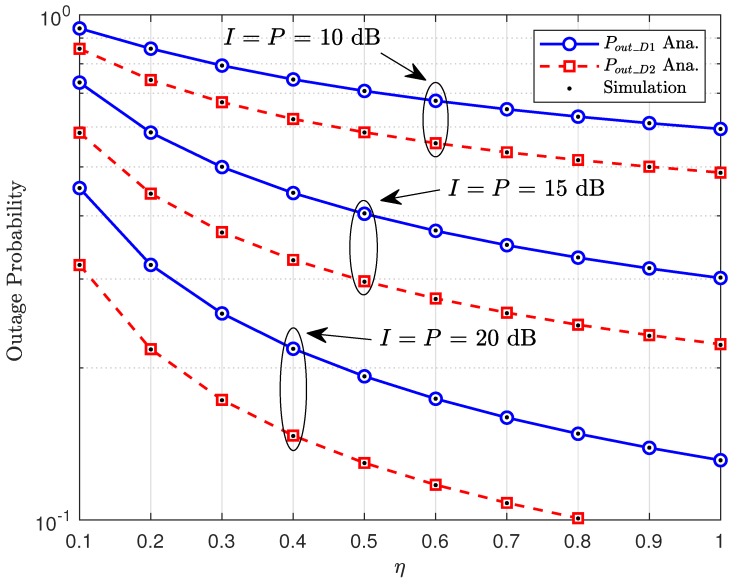
Impact of percentage of harvested power at RSU on outage performance with different cases of I,P.

Figure 4 illustrates that such outage performance is affected by the amount of harvested energy at RSU. When η is small, it means the amount of harvested energy is reduced. This situation makes the outage performance becomes worse value. However, we only see larger performance gaps among three cases η=0.3,0.5,0.9 at a low region of SNR. It is further observed that the outage probability decreases significantly as this energy efficiency η increases as in Figure 5. This figure confirms the higher power of primary users contributing to enhanced performance of secondary networks. Therefore, the advantage of the cognitive radio and energy harvesting schemes benefit V2X to guarantee operation of both networks (secondary and primary networks).

**Figure 6:**
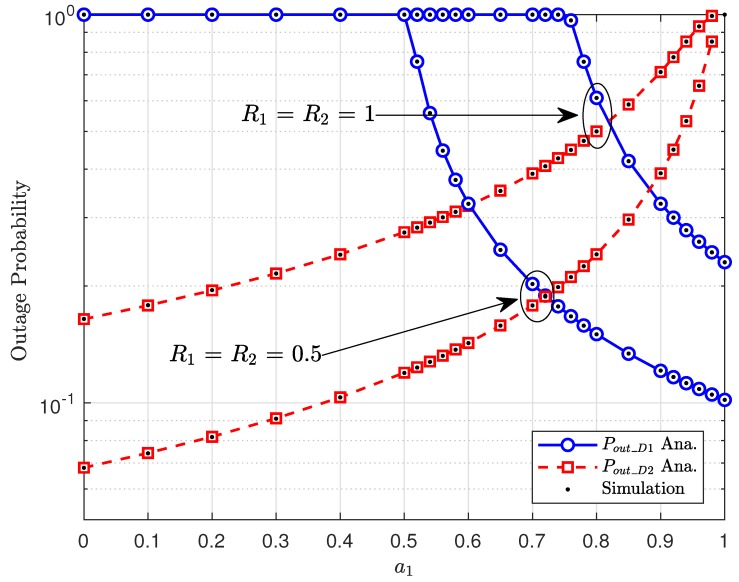
Impact of power allocation factors on the performance of the NOMA-based V2X system.

Although the number of RSUs and the amount of harvested energy are crucial parameters to the system performance, from Figure 6, power allocation factors $a_{1},a_{2}$ significantly change the outage performance. As in the previous figures, $D_{2}$ shows better performance compared with that of $D_{1}$; however, increasing $a_{1}$ contributes to contrasting trends. For example, the outage performance of $D_{1}$ is better than that of $D_{2}$ as $a_{1}>0.6$ and $R_{1}=R_{2}=0.5$; the outage performance of $D_{1}$ is better than that of $D_{2}$ as $a_{1}>0.83$ and $R_{1}=R_{2}=1$.

## 5. Conclusions

This paper presented theoretical analysis and the performance comparison of the CR-enabled V2X-NOMA system which adopts DF forwarding strategy, RSU selection, and energy harvesting. Particularly, this paper derived closed-form expressions of outage probability, based on which various comparisons for outage probability of two vehicles were presented. Furthermore, based on the performance analysis, we studied that a greater number of RSUs and a larger amount of harvested power result in better performance in the context of various system metrics.

## Appendix A

**Proof.** **Proof of** *A*_1_ Based on (1), $A_{1}$ can be computed as

(A1) $$A_{1}=\underset{A_{11}}{\underset{︸}{\mathrm{Pr}\left\{{\left|h_{SR_{k}}\right|}^{2}<\frac{\delta }{P},{\left|h_{SP}\right|}^{2}<\frac{I}{P}\right\}}}+\underset{A_{12}}{\underset{︸}{\mathrm{Pr}\left\{\frac{{\left|h_{SR_{k}}\right|}^{2}}{{\left|h_{SP}\right|}^{2}}<\frac{\delta }{I},{\left|h_{SP}\right|}^{2}>\frac{I}{P}\right\}}}$$

Then, $A_{11}$ and $A_{12}$ can be obtained as, respectively,

(A2) $$\begin{array}{cc} A_{11} & =\mathrm{Pr}\left\{{\left|h_{SR_{k}}\right|}^{2}<\frac{\delta }{P},{\left|h_{SP}\right|}^{2}<\frac{I}{P}\right\}\\ \; & =\left[1-\sum_{k=1}^{K}\left(\begin{array}{c} K\\ k \end{array}\right){\left(-1\right)}^{k-1}\left(\exp\left(-\frac{\delta k}{P\Omega_{SR}}\right)\right)\right]\\ \; & \times \left(1-\exp\left(-\frac{I}{P\Omega_{SP}}\right)\right) \end{array}$$

In addition, $A_{12}$ is given by

(A3) $$\begin{array}{cc} A_{12} & =\mathrm{Pr}\left\{\frac{{\left|h_{SR_{k}}\right|}^{2}}{{\left|h_{SP}\right|}^{2}}<\frac{\delta }{I},{\left|h_{SP}\right|}^{2}>\frac{I}{P}\right\}\\ & =\frac{1}{\Omega_{SP}}\int_{\frac{I}{P}}^{\infty }\left(1-\sum_{k=1}^{K}\left(\begin{array}{c} K\\ k \end{array}\right){\left(-1\right)}^{k-1}\left(\exp\left(-\frac{\delta kx}{I\Omega_{SR}}\right)\right)\right)\exp\left(-\frac{x}{\Omega_{SP}}\right)dx\\ & =\exp\left(-\frac{I}{P\Omega_{SP}}\right)-\frac{I\Omega_{SR}}{I\Omega_{SR}+\delta k\Omega_{SP}}\sum_{k=1}^{K}\left(\begin{array}{c} K\\ k \end{array}\right){\left(-1\right)}^{k-1}\left(\exp\left(-\left(\frac{\delta k\Omega_{SP}+I\Omega_{SR}}{I\Omega_{SR}\Omega_{SP}}\right)\frac{I}{P}\right)\right). \end{array}$$

 □

## Appendix B

**Proof.** **Derivation of** *A*_22_ It can be rewritten $A_{22}$ as

(A4) $$\begin{array}{cc} A_{22} & =\mathrm{Pr}\left\{{\left|h_{SR_{k}}\right|}^{2}>\frac{\delta }{P},{\left|h_{R_{k}D_{1}}\right|}^{2}<\frac{\delta {\left|h_{R_{k}P}\right|}^{2}}{I},{\left|h_{R_{k}P}\right|}^{2}>\frac{I}{\eta \left(P{\left|h_{SR_{k}}\right|}^{2}-\delta \right)}\right\}\\ \; & =\int_{\frac{\delta }{P}}^{\infty }f_{{\left|h_{SR_{k}}\right|}^{2}}\left(x\right)dx\int_{\frac{I}{\eta \left(Px-\delta \right)}}^{\infty }f_{\delta {\left|h_{R_{k}P}\right|}^{2}}\left(y\right)F_{{\left|h_{R_{k}D_{1}}\right|}^{2}}\left(\frac{\delta y}{I}\right)dy \end{array}$$

Then, $A_{22}$ can be further computed by

(A5) $$A_{22}=\sum_{k=1}^{K}\left(\begin{array}{c} K\\ k \end{array}\right)\frac{k{\left(-1\right)}^{k-1}}{\Omega_{SR}}\int_{\frac{\delta }{P}}^{\infty }\left(e^{-\frac{kx}{\Omega_{SR}}}\right)dx\left(e^{-\frac{I}{\Omega_{RP}\eta \left(Px-\delta \right)}}-\frac{I\Omega_{RD_{1}}}{\delta \Omega_{RP}+I\Omega_{RD_{1}}}e^{-\left(\frac{\delta \Omega_{RP}+I\Omega_{RD_{1}}}{\Omega_{RD_{1}}\Omega_{RP}\eta \left(Px-\delta \right)}\right)}\right)$$

Thus, the closed-form expression of $A_{22}$ can be obtained as

(A6) $$\begin{array}{cc} A_{22} & =2\sum_{k=1}^{K}\left(\begin{array}{c} K\\ k \end{array}\right){\left(-1\right)}^{k-1}e^{-\frac{k\delta }{\Omega_{SR}P}}\left(\sqrt{\frac{Ik}{\Omega_{RP}\Omega_{SR}P\eta }}K_{1}\left(2\sqrt{\frac{Ik}{\Omega_{RP}\Omega_{SR}P\eta }}\right)\right.\\ \; & -\left.\frac{I\Omega_{RD_{1}}}{\delta \Omega_{RP}+I\Omega_{R_{k}D_{1}}}\sqrt{\frac{\left(\delta \Omega_{RP}+I\Omega_{RD_{1}}\right)k}{\Omega_{RD_{1}}\Omega_{RP}\Omega_{SR}P\eta }}K_{1}\left(2\sqrt{\frac{\left(\delta \Omega_{RP}+I\Omega_{RD_{1}}\right)k}{\Omega_{RD_{1}}\Omega_{RP}\Omega_{SR}P\eta }}\right)\right) \end{array}$$

It completes the proof. □
